# The Water Drinker Three Centuries Ago

**Published:** 1895-11-23

**Authors:** 


### THE WATER-DRINKER THREE CENTURIES AGO.

In the good old days the man who chose either from taste or principle to abstain from strong drink met with scant tolerance from his neighbours. His was not a large following, he did not rush into print, and his very existence might be questioned were it not for the shafts of scorn launched against him by the writers of the period. It is possible, however, from the collective abuse of his adversaries, to form a tolerably clear picture of his position in Shakespeare's day. The singularity of his opinions developed in him a grim obstinacy, which fully accounts for the vehement enmity of the vulgar, and the petulant disapprobation of the learned. He was marked out as an "odd abstemious, one in a thousand perchance, degenerate, and of a doggish nature, for dogs of nature do abhor wine." His presence at festivals was shunned, for the common verdict declared "a man cannot make him laugh, but that's no marvel, he drinks no wine." So in Harrington's rhymed version of the "Regimen Sanitatis Salerni," he is doomed to solitude—

Some to drink only water are assigned,
But such by our consent shall dine alone.

And his looks gave some show of reason to the common prejudice, for it may readily be believed that in days when tea, coffee, and cocoa were unknown, and water was considered little short of poison, the abstemious may have come off rather badly. The wine-bibber claimed that "extenuate, withered bodies by wine were caused to be fair, fresh, plump, and fat; old and infirm to be young and sound, wheneas water or small-beer drinkers look like apes rather than men."

The abstemious, nevertheless, all honour to him, held his ground in the teeth of much opposition and real privation, uncomfortably quenching his thirst by means of such non-alcoholic devices as the times afforded, and even daring in some cases to taste if not to depend on the forbidden element itself.

It needed a very bold man to resist the medical testimony of the period against water drinking. Few writers can be found to say a good word for it. One or two only are concerned to maintain that, when begun in early life, it may be pretty freely drunk with impunity, and they quote the curious instance given by Sir Thomas Elyot in his "Castle of Health," 1541, of the Cornish men, "many of the poorer sort, which never, or very seldom, drink any other drink, be notwithstanding strong of body, and like and live well until they be of great age." Thomas Cogan, the medical schoolmaster of Manchester fame, confessed in his "Haven of Health," 1589, designed for the use of students, that he knew some who drink cold water at night or fasting in the morning without hurt; and Dr. James Hart, writing about fifty years later, could even claim among his acquaintance "some honourable and worshipful ladies who drink little other drink, and yet enjoy more perfect health than most of them that drink of the strongest." The phenomenon was undeniable, but the natural inference was none the less to be resisted. Sir Thomas Elyot himself is very certain, in spite of the Cornish men, that "there be in water causes of divers diseases, as of swelling of the spleen and liver." He complains oddly also that "it flitteth and swimmeth," and concludes that "to young men, and them that be of hot complexion, it doeth less harm, and sometimes it profiteth, but to them that are feeble, old, and melancholy, it is not convenient."

"Water is not wholesome cool by itself for an Englishman" was the verdict of Andrew Borde—monk, physician, bishop, ambassador, and writer on sanitation—as the result of a life's experience. And to quote again the "Englishman's Doctor"—

Both water and small beer, we make no question,
Are enemies to health and good digestion.

But the most formal indictment against water is that of Venner, who, writing in 1622, ponderously pronounces "to dwellers in cold countries it doth very greatly deject their appetites, destroy the natural heat and overthrow the strength of the stomach, and consequently confounding the concoction, is the cause of crudities, fluctuations, and windiness in the body."

Still there were situations in which a man might be compelled, for want of something better to drink, to risk all these dangers. In such a contingency there were many precautions to be urged on the unlucky water-drinker, and some go far to justify the common prejudice. You are, in any case, to see that you seeth it gently first, and that is best "whereof cometh least skim or froth when it doth boil." If putrid you may revive it with oil of sulphur or aqua-vitæ. Good water might be known by its weight, "that which is lightest is best," by its readiness to boil, and by "drenching linen cloths in it and laying the same to dry, that which is soonest dry showeth best water." Rain water was esteemed the best of all, and next to it was "running water, the which doth swiftly run from east to west upon stones or pebbles."

It is curious that the germicidal action of the sun, only quite recently established as a scientific fact, was then clearly recognised, and the water-drinker was cautioned "against the use of well water to which the sun hath no reflection." Doctor Hart notes various methods of purification in common use, such as boiling it down to half or a third of the original quantity, straining it through clean linen cloths, boiling it with sand, or with coral beaten to powder, to correct its bitterness; and, lastly, by the costly process of distillation. "Some used to add sugar to it, and is most practised by the female sex," but this addition the doctor thinks useless and even pernicious. And especially he cautions his readers against drinking of the "River of Ware, conveyed to the noble City of London through leaden pipes," having a not altogether ungrounded suspicion that the water may thereby become impregnated with poisonous particles.

Good water then was hard to get, its use was prohibited altogether in winter; and even in hot weather, when least harmful, it was emphatically decreed that "it ought seldom to be drunk." Yet, within half a century from these learned dicta the water-drinker had his revenge, and the despised element was exalted into the position of a panacea.

